# Silencing of circCacna1c Inhibits ISO-Induced Cardiac Hypertrophy through miR-29b-2-5p/NFATc1 Axis

**DOI:** 10.3390/cells12121667

**Published:** 2023-06-19

**Authors:** Peilei Lu, Danyu Zhang, Fan Ding, Jialu Ma, Yang K. Xiang, Meimi Zhao

**Affiliations:** 1 Department of Pharmaceutical Toxicology, School of Pharmacy, China Medical University, Shenyang 110122, China; lupeilei95@hotmail.com (P.L.); zhangdanyu327@163.com (D.Z.); dingfan@xjtu.edu.cn (F.D.); 13925219615@163.com (J.M.); 2 Department of Pharmacology, University of California at Davis, Davis, CA 95616, USA

**Keywords:** circRNA, circCacna1c, miR-29b-2-5p, NFATc1, cardiac hypertrophy

## Abstract

Pathological cardiac hypertrophy is one of the notable causes of heart failure. Circular RNAs (circRNAs) have been studied in association with cardiac hypertrophy; however, the mechanisms by which circRNAs regulate cardiac hypertrophy remain unclear. In this study, we identified a new circRNA, named circCacna1c, in cardiac hypertrophy. Adult male C57BL/6 mice and H9c2 cells were treated with isoprenaline hydrochloride (ISO) to establish a hypertrophy model. We found that circCacna1c was upregulated in ISO-induced hypertrophic heart tissue and H9c2 cells. Western blot and quantitative real-time polymerase chain reaction showed that silencing circCacna1c inhibited hypertrophic gene expression in ISO-induced H9c2 cells. Mechanistically, circCacna1c competitively bound to miR-29b-2-5p in a dual-luciferase reporter assay, which was downregulated in ISO-induced hypertrophic heart tissue and H9c2 cells. MiR-29b-2-5p inhibited the nuclear factor of activated T cells, cytoplasmic, calcineurin-dependent 1 (NFATc1) to control hypertrophic gene expression. After silencing circCacna1c, the expression of miR-29b-2-5p increased, which reduced hypertrophic gene expression by inhibiting NFATc1 expression. Together, these experiments indicate that circCacna1c promotes ISO-induced pathological hypertrophy through the miR-29b-2-5p/NFATc1 axis.

## 1. Introduction

Pathological cardiac hypertrophy is one of the notable causes of heart failure and a significant threat to human health and quality of life [1]. Globally, the incidence of heart failure is estimated to be approximately 64.3 million. Moreover, the five-year survival rate of patients who have experienced heart failure is approximately 57% [2,3]. Cardiac hypertrophy is an adaptive response of the heart to stimuli and can be generally divided into physiological or pathological hypertrophy in different ways, depending on the stimulus [4]. At the cellular level, cardiomyocyte enlargement is a major pathological feature of cardiac hypertrophy, including increased protein synthesis and overexpression of hypertrophic genes, including atrial natriuretic peptide (ANP), brain natriuretic peptide (BNP), etc. [5]. Evidence indicates that calcium signaling plays a crucial role in the development and progression of pathological hypertrophy [6,7,8]. Nuclear factor of activated T cells (NFAT), as a member of cardiac transcription factors, can reactivate fetal genes and participate in the hypertrophy signaling pathway [9,10,11].

Noncoding RNAs are emerging regulators in pathological hypertrophy, including microRNAs (miRNAs), long noncoding RNAs, and circular RNAs (circRNAs). Different from linear RNA, circRNAs have a covalently closed-loop structure, which determines its structural stability, and are resistant to exonucleases, such as ribonuclease R (RNase R). CircRNAs also have a longer half-life, which suggests that it may be used as a biomarker of disease [12,13,14]. The main functions of circRNA have been discovered, such as regulating the expression of their parental gene and acting as miRNA sponges [15]. Over the last decade, an increasing number of circRNAs have been identified in pathological hypertrophy, including circRNA_000203 [14], circSlc8a1 [15], heart-related circRNA (HRCR) [16], circHIPK3 [17,18], CircSIRT1 [19], Circ_0001052 [20], etc. These circRNAs act as miRNA sponges to regulate gene expression, thereby affecting pathological hypertrophy.

Conversely, miRNAs are a class of small noncoding RNAs with a length of approximately 18–24 nucleotides, which can inhibit the translation or degradation of messenger RNAs (mRNAs) by targeting the 3′-untranslated region (3′-UTR) of their target mRNAs [21,22,23]. Studies have demonstrated the physiological and pathogenic roles of miRNAs in cardiac hypertrophy, such as miR-1 [23], miR-133 [24], and miR-155 [25].

Here, we identified and characterized a novel circRNA encoded by Cacna1c, named circCacna1c. We explored the role of circCacna1c in the regulation of cardiac hypertrophy by targeting the miR-29b-2-5p/NFATc1 axis in the heart.

## 2. Materials and Methods

### 2.1. Animals and ISO-Induced Animal Model

Adult male C57BL/6 mice (8 weeks old) were purchased from Sipeifu Biotechnology (Beijing, China). The mice were randomly divided into the control group (*n* = 3) and the model group (*n* = 3). An animal model of cardiac hypertrophy was established by subcutaneous injection of isoprenaline hydrochloride (ISO, TCI, Lot. MI5VB-DI, Tokyo, Japan) once daily at 2 mg·kg^−1^·d^−1^ for 14 days [26]. At the end of the injection, mice were anesthetized intraperitoneally with sodium pentobarbital and sacrificed to harvest the heart tissue. Here, we took the left ventricle of the heart (including the interventricular wall) and cut it into upper and lower parts. The upper part was soaked in 4% paraformaldehyde for HE staining, and the lower part was stored at −80 °C for circular RNA microarray.

All experiments were carried out in accordance with the guidelines for Animal Experimentation of China Medical University and followed NIH guidelines.

### 2.2. Circular RNA Microarray and Expression Profiling Analysis

A circular RNA (circRNA) microarray was performed according to Arraystar’s standard protocols (Arraystar Inc., Rockville, MD, USA) [27,28]. Briefly, total RNAs were digested with Rnase R (Epicentre, Inc., Madison, WI, USA) to remove linear RNAs and enrich circular RNAs. Then, the enriched circular RNAs were amplified and transcribed into fluorescent cRNA, utilizing a random priming method (Arraystar Super RNA Labeling Kit; Arraystar). The labeled cRNAs were purified using an RNeasy Mini Kit (Qiagen, Hilden, Germany). The concentration and specific activity of the labeled cRNAs were determined by NanoDrop ND-1000. The labeled cRNAs were hybridized onto the Arraystar Mouse circRNA Arrays (8 × 15 K, Arraystar) and incubated for 17 h at 65 °C in an Agilent Hybridization Oven. The hybridized arrays were washed, fixed, and scanned using the Agilent Scanner G2505C. Scanned images were imported into Agilent Feature Extraction software for raw data extraction. The statistical significance of the difference may be conveniently estimated by *t*-test. CircRNAs with fold changes (FC) ≥ 1.5 and *p* < 0.05 were considered to be significantly differentially expressed. 

Kyoto Encyclopedia of Genes and Genomes (KEGG) pathway enrichment analysis of the host genes of differently expressed circRNAs and their target mRNAs were performed using the DAVID (https://david.ncifcrf.gov/, accessed on 21 July 2020) and Metascape websites (http://metascape.org/, accessed on 21 July 2020).

### 2.3. Fluorescence In Situ Hybridization

Heart tissues were fixed in 4% paraformaldehyde, embedded by paraffin, and sliced through the slicer. The fixed tissues were incubated with FAM(488)-labeled RNA probes targeting circCacna1c in a hybridization buffer overnight at 42 °C. Then, the tissues were incubated with DAPI staining. Images were captured using a fluorescence microscope (Nikon, Tokyo, Japan). The circCacna1c probe sequence was 5′-FAM-CCCATAGTTGGAACCAGGTTGGAGTTGG-3′ (ServiceBio, Wuhan, China).

### 2.4. Cell Culture and Transfection

The H9c2 cells (ATCC, Manassas, VA, USA) were cultured in high-glucose Dulbecco’s modified Eagle’s medium (HyClone, Cat# SH30022.01, Logan, UT, USA) containing 10% fetal bovine serum and 1% penicillin/streptomycin at 37 °C in an incubator with 5% CO_2_. For the ISO-induced cell model, the H9c2 cells were incubated with 30 μM ISO in a serum-free medium for 36 h.

Small interfering RNA (siRNA) oligonucleotides specific to circCacna1c and negative control siRNA (si-NC) were designed and synthesized by RiboBio (Guangzhou, China). The siRNA sequence for circCacna1c was 5′-UCCCAUAGUUGGAACCAGG-3′. The mmu-miR-29b-2-5p mimic (miR-mimic) and their corresponding negative controls (NC-mimic) were purchased from RiboBio. The sequence for miR-mimic was 5′-CUGGUUUCACAUGGUGGCUUAGAUU-3′. The siRNAs or miRNA mimics were transfected into H9c2 cells using the riboFECTTM CP transfection reagent (RiboBio, Cat# C10511-1, Guangzhou, China), according to the manufacturer’s instructions.

### 2.5. RNase R Treatment

RNase R digestion was performed to test the stability of circCacna1c. Total RNA was extracted from H9c2 cells using TRIzol reagent (Invitrogen, Cat# 15596-026, Waltham, MA, USA). Then, total RNA (1 μg) in 1× RNase R reaction buffer at a total volume of 10 μL was incubated for 15 min at 37 °C with 1 U/μg of RNase R (Epicentre Biotechnologies, Cat# RNR07250, Madison, WI, USA) or DNase/RNase-Free water; subsequently, the enzymes were inactivated at 70 °C for 10 min.

### 2.6. Quantitative Real-Time Polymerase Chain Reaction

Total RNA was extracted from H9c2 cells or heart tissue using TRIzol reagent (Invitrogen, Cat# 15596-026, USA). For circRNA and mRNA expression analysis, cDNAs were synthesized from total RNA using the PrimeScript™ RT Master Mix (Takara, Cat# RR036A, Osaka, Japan). The expression levels of the target genes were determined using a SYBR Green qPCR kit (Bimake, Cat# B21202, Houston, TX, USA) with QuantStudio™ 1 System (Applied Biosystems, Carlsbad, CA, USA). GAPDH served as the internal reference. The following primer sequences were used:

CircCacna1c,

forward 5′-GCAATCTATATTCCCTTTCCG-3′,

reverse 5′-GAGTAGGGATGTGCTCGGG-3′;

Line Cacna1c,

forward 5′-GCCTCGAAGTTGGGAGAACA-3′,

reverse 5′-TGTGTGGGAGTCAATGGAGC-3′;

ANP,

forward 5′-CTGGGACCCCTCCGATAGAT-3′,

reverse 5′-GTCAATCCTACCCCCGAAGC-3′;

BNP,

forward 5′-ACAATCCACGATGCAGAAGCT-3′,

reverse 5′-GGGCCTTGGTCCTTTGAGA-3′;

NFATc1,

forward 5′-CACCTTGGCACCTCTTTCATAGTCAG-3′,

reverse 5′-TCTTGGGCTGTTCAACTTCCTTCAC-3′;

GAPDH,

forward 5′-CAACGACCCCTTCATTGACC-3′,

reverse 5′-GAAGACGCCAGTAGACTCCA-3′.

For miRNA expression analysis, mmu-miR-29b-2-5p and U6 were reverse-transcribed to cDNA using a stem-loop primer by riboSCRIPTTM Reverse Transcription Kit (RiboBio, Cat# C11027-2, China) and specific Bulge-LoopTM miRNA RT primers (RiboBio, China). The U6 gene served as an endogenous control. These data were analyzed using the 2^−ΔΔCT^ method. ΔCT was the mean CT value of each sample minus the corresponding mean CT value of GAPDH (internal reference). ΔΔCT was the ΔCT value of each sample minus the ΔCT of the first one control sample. Each group conducted 3 independent experiments as 3 samples.

### 2.7. TRITC-Phalloidin Staining

To assess the cell size of the H9c2 cells, the cultured H9c2 cells were fixed using a 4% formaldehyde solution dissolved in PBS for 10 min at room temperature and were permeabilized with a 0.5% Triton X-100 solution for 5 min. Afterwards, they were protected from light and incubated at room temperature for 30 min using 150 nM TRITC-Phalloidin (Solarbio, Cat# CA1610, Beijing, China). Finally, the cell nuclei were stained using DAPI. The pictures were captured with an inverted fluorescent microscope (Leica, Wetzlar, Germany).

### 2.8. Western Blot

H9c2 cells were lysed with RIPA lysis buffer containing protease inhibitors on ice for 30 min and then ultrasound for 5 s; cells debris was removed by centrifugation at 12,000 rpm for 15 min at 4 °C. Cell supernatant and SDS-loading buffer were mixed and boiled in boiling water for 5 min. Separate equal amounts of protein by SDS-PAGE were then transferred to polyvinylidene fluoride (PVDF) membranes (Millipore, Burlington, MA, USA). The membranes were blocked with 5% BSA in 0.05% Tween 20-PBS at room temperature for 1 hr. Primary antibodies against ANP (Abmart, Cat# TD6497, Shanghai, China), BNP (Wanleibio, Cat# WL02126, Shenyang, China), NFATc1 (Abmart, Cat# TD6446, China), and β-actin (Bioworld, Cat# BS6007M, Dublin, OH, USA) were used to blot specific proteins at 4 °C overnight. The goat anti-mouse IgG (ABclonal, Cat# AS003, Woburn, MA, USA) and goat anti-rabbit IgG (Cell Signaling Technology, Cat# 7074P2, Danvers, MA, USA) secondary antibodies were used to reveal the primary antibodies using the MicroChemi Gel Imaging System (DNR, Modi’in-Maccabim-Re’ut, Israel) by applying the ECL substrate. The optical density of the bands was analyzed with Image J software (Version 1.53t, NIH, Bethesda, MD, USA). The data were expressed as the ratio of the intensity of interested protein to the intensity of β-actin.

### 2.9. Laser Confocal Measurement for [Ca^2+^]_i_

The H9c2 cells were loaded with 5μM Fluo-4/AM (Beyotime, Cat# S1060, Shanghai, China) for 30 min at 37 °C, then washed twice to remove the extracellular dye. The culture dishes containing cells were mounted on the stage of a laser confocal microscope NikonA1R (Nikon, Tokyo, Japan). NIS-Elements (Version 5.01.00, Nikon, Tokyo, Japan) was used for hardware control, image acquisition, and image analysis. Green fluorescence was excited at 488 nm. The sampling rate was set to one frame every 5 s with a typical exposure time of 50 ms. Images were corrected for background, and regions of interest were selected manually. [Ca^2+^]_i_ was expressed as the ratio of the fluorescence intensity of Fluo-4/AM after drug administration (F_max_) to baseline fluorescence intensity (F_0_).

### 2.10. Dual-Luciferase Reporter Assay

The recombinant luciferase reporter plasmids containing the potential mmu-miR-29b-2-5p binding site sequences in circCacna1c and the plasmids that mutated the binding sites were constructed by Genechem (Shanghai, China). HEK293T cells were seeded into 96-well plates (4 × 10^3^ cells/well) and cultured for 24 h, then co-transfected with 100 ng of circCacna1c or mutated plasmids and 4 pmol of mmu-miR-29b-2-5p mimic or NC-mimic using Lipo8000^TM^ Transfection Reagent (Beyotime, Cat# C0533, Shanghai, China) for 48 h. Finally, the luciferase activities were detected with the Dual-Luciferase^®^ Reporter Assay System (Promega, Cat# E1910, Madison, WI, USA) using a multifunctional microplate reader (BioTek, Cat# H1MD, Burlington, VM, USA). To normalize for transfection efficiency, the firefly luciferase activity values were normalized to the Renilla luciferase activity values.

### 2.11. Statistics Analysis

All data are expressed as the mean ± standard error of mean (SEM). Data analyses were carried out using IBM SPSS Statistics for Windows, version 25 (IBM Corp., Armonk, NY, USA). Differences were evaluated by unpaired *t*-test for two groups or one-way ANOVA with LSD’s post-test for multiple groups. A significant difference was defined as *p* < 0.05.

## 3. Results

### 3.1. CircRNA Profiling in ISO-Induced Hypertrophic Heart Tissue

**Figure 1:**
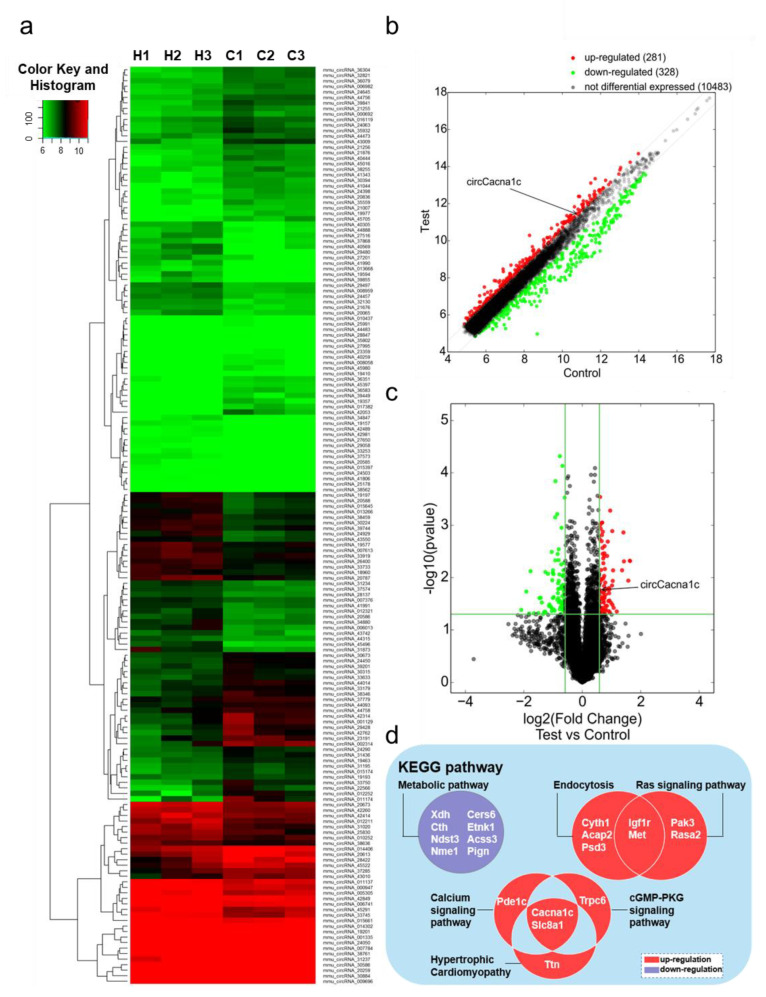
Gene expression analysis in ISO-induced hypertrophic heart tissue by circRNA microarray. (**a**) Hierarchical clustering analysis of circRNA expression profiles in ISO and control groups (H1–3, ISO group; C1–3, control group). (**b**) Scatter plot analysis of circRNA abnormalities in ISO group and control groups (the dots outside the green lines indicate upregulated or downregulated differential circRNAs). (**c**) Volcano plot analysis of abnormal expression of circRNAs between the two groups (the red data blocks indicate differential circRNAs). (**d**) KEGG analysis of the host genes of circRNAs differently expressed. circRNA: circular RNA; ISO: isoprenaline hydrochloride; KEGG: Kyoto Encyclopedia of Genes and Genomes.

To investigate the differential expression levels of circRNAs in mouse cardiac hypertrophy, we successfully constructed an ISO-induced myocardial hypertrophy mouse model (Figure S1 and Table S1). Simultaneously, the corresponding heart tissues were subjected to a circRNA microarray. The heat map shows that a total of 11,092 circRNAs were detected (Figure 1a). Scatter plots (Figure 1b) and volcano plots (Figure 1c) reveal that 166 circRNAs were differentially expressed with a combination of statistical significance (FC ≥ 1.5, *p* < 0.05), including 81 upregulated circRNAs and 85 downregulated circRNAs. 

KEGG pathway analysis showed that upregulated genes were mainly enriched in five signaling pathways, namely hypertrophic cardiomyopathy (*Slc8a1*, *Pde1c*, *Cacna1c*), endocytosis (*Igf1r*, *Cyth1*, *Acap2*, *Met*, *Psd3*), the Ras signaling pathway (*Igf1r*, *Pak3*, *Met*, *Rasa2*), the cGMP-PKG signaling pathway (*Slc8a1*, *Cacna1c*, *Trpc6*), and the calcium ion signaling pathway (*Slc8a1*, *Cacna1c*, *Ttn*). The downregulated genes were only enriched in the metabolic signaling pathway (*Xdh*, *Cth*, *Ndst3*, *Nme1*, *Cers6*, *Etnk1*, *Ascc3*, *Pign*). *Cacna1c* and *Slc8a1* were both enriched in three different pathways (Figure 1d). The host gene *Slc8a1*, which encodes Na^+^/Ca^2+^ exchangers 1(NCX1), regulates cellular Ca^2+^ homeostasis and affects heart failure. Meanwhile, circSlc8a1 has been shown to regulate cardiac hypertrophy [15]. Therefore, we conjectured the circRNA encoded by *Cacna1c*, thereby named circCacna1c, plays a role in pathological hypertrophy.

### 3.2. CircCacna1c Expression in ISO-Induced Hypertrophic Tissue and Cells

**Figure 2:**
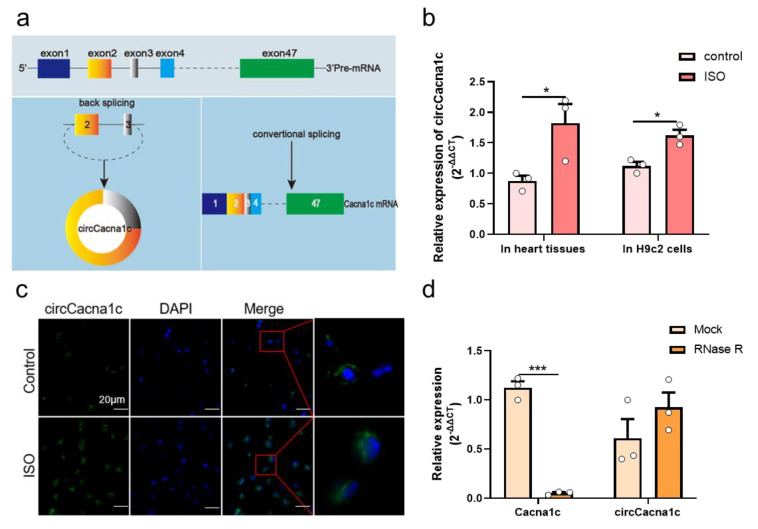
CircCacna1c expression in ISO-induced hypertrophic tissue and H9c2 cells. (**a**) Biogenesis of circCacna1c (**left**) and linear Cacna1c mRNA (**right**). (**b**) Detection of the expression of circCacna1c in ISO-induced heart tissue and H9c2 cells by quantitative real-time polymerase chain reaction assays. (**c**) RNA fluorescence in situ hybridization of circCacna1c in heart tissue. circCacna1c is shown in green and nuclei were stained with DAPI (blue). Scale bar: 20 μm. (**d**) circCacna1c and Cacna1c mRNA levels treatment were measured by quantitative real-time polymerase chain reaction assay in H9c2 cells with (RNase R group) or without RNase R (Mock group). *T*-tests were used for statistical analysis. Data are shown as mean ± SEM. *n* = 3. * *p* < 0.05, *** *p* < 0.001. ISO: isoprenaline hydrochloride; RNase R: ribonuclease R.

CircCacna1c is located on mouse chromosome 6 at position 119002618-119007533 and is 4915 bases long in the genome, whereas the sheared mature circRNA is 428 bases in length. It is formed by the cyclization of the second and third exons of the *Cacna1c* antisense chain (Figure 2a). The circCacna1c nucleic acid sequence is relatively conserved, with a similarity of 96% between mouse and rat species (Table S2) and 89% between mouse and human species (Table S3). In a mouse model of cardiac hypertrophy induced by ISO infusion, the expression levels of circCacna1c were upregulated relative to the control tissue. Additionally, the expression level of circCacna1c was also upregulated in H9c2 cells after treatment with ISO for 36 h (Figure 2b). Subsequently, RNA fluorescence in situ hybridization demonstrated that circCacna1c is mainly located in the cytoplasm (Figure 2c). Moreover, we performed an RNase R resistance assay on total RNA isolated from H9c2 cells, followed by quantitative real-time polymerase chain reaction. The results show that circCacna1c was resistant to RNase R digestion compared with Cacna1c mRNA (Figure 2d).

### 3.3. Silencing circCacna1c Inhibits Hypertrophic Gene Expression in ISO-Induced H9c2 Cells

**Figure 3:**
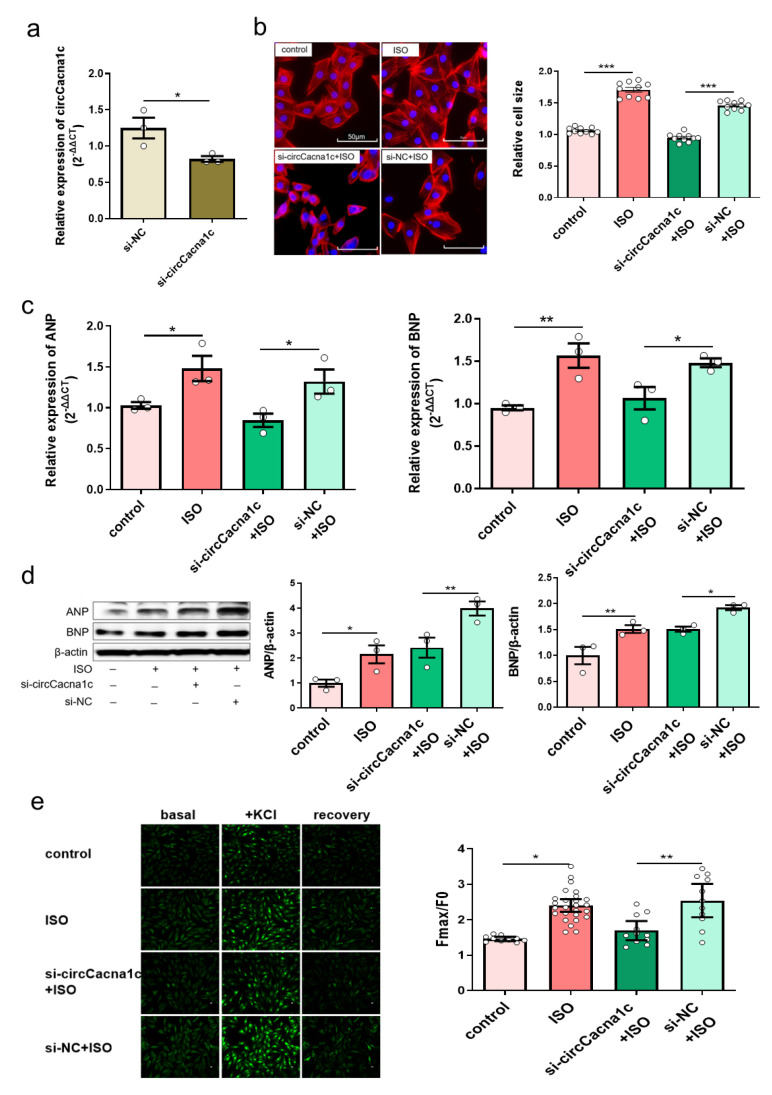
Silencing circCacna1c inhibits hypertrophic gene expression in ISO-induced H9c2 cells. (**a**) siRNA knockdown efficiency against circCacna1c by quantitative real-time polymerase chain reaction assay. *T*-tests were used for statistical analysis, *n* = 3. (**b**) H9c2 cells expressing circCacna1c and control siRNA were treated with ISO for 36 h. The morphology of ISO-induced H9c2 cells was examined with TRITC-Phalloidin staining assay. The scale bar is 50 μm. One-way ANOVA was used for statistical analysis, *n* = 10. (**c**,**d**) The mRNA and protein expression of ANP and BNP were examined in ISO-induced H9c2 cells, respectively. One-way ANOVA was used for statistical analysis, *n* = 3. (**e**) Change in intracellular calcium concentration induced by KCl. One-way ANOVA was used for statistical analysis, *n* = 10–26. Data are shown as mean ± SEM. * *p* < 0.05, ** *p* < 0.01, *** *p* < 0.001. ISO: isoprenaline hydrochloride; ANP: atrial natriuretic peptide; BNP: brain natriuretic peptide.

We next examined whether the progression of cardiac hypertrophy could be modulated by silencing circCacna1c. siRNA specific for circCacna1c (si-circCacna1c) and negative control siRNA (si-NC) was transfected to H9c2 cells for 24 h, and the knockdown efficiency of si-circCacna1c was assessed, which showed that circCacna1c expression levels were reduced successfully (Figure 3a). At 24 h post-transfection, H9c2 cells were stimulated with 30 μM ISO for 36 h. Cell sizes were reduced after silencing circCacna1c relative to the si-NC control in TRITC-Phalloidin staining (Figure 3b). Meanwhile, the expression of hypertrophic genes ANP and BNP was decreased in ISO-induced H9c2 cells with knockdown of circCacna1c when compared with the si-NC group (Figure 3c). Consistently, the protein expression of ANP and BNP was also reduced (Figure 3d). Meanwhile, the KCl-induced intracellular calcium was markedly increased in H9c2 after 36 h of treatment with ISO compared with the control. Pre-treatment with si-circCacna1c, but not si-NC, attenuated intracellular calcium in ISO-treated H9c2 cells (Figure 3e). These results indicate that circCacna1c knockdown inhibits the level of intracellular calcium and hypertrophic gene expression after chronic exposure to ISO in H9c2 cells.

### 3.4. CircCacna1c Competitively Binds to miR-29b-2-5p

**Figure 4:**
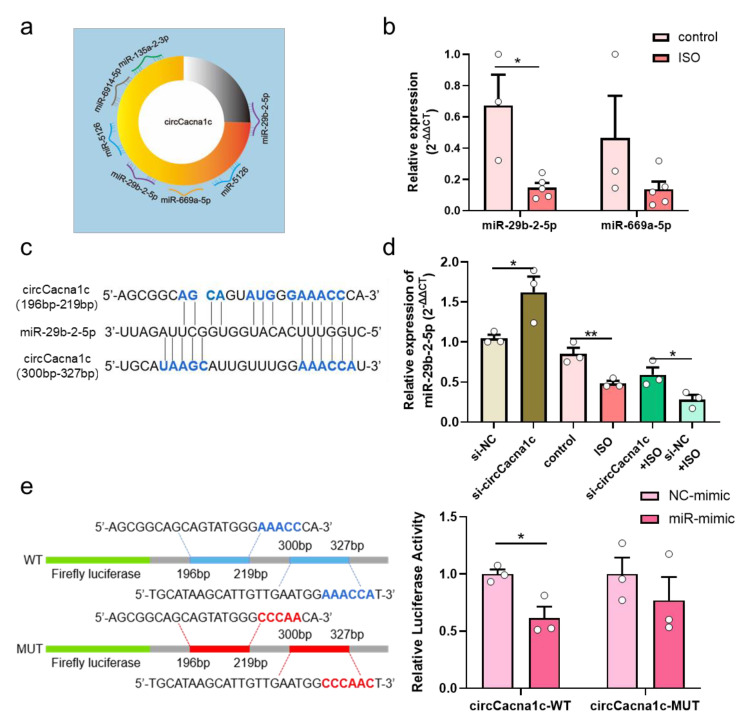
CircCacna1c competitively binds to miR-29b-2-5p. (**a**) Target miRNAs of circCacna1c predicated by Arraystar’s homemade miRNA target prediction software based on TargetScan and miRanda. (**b**) The expression of miR-29b-2-5p and miR-669a-5p in ISO-induced heart tissue. *T*-test was used for statistical analysis, *n* = 3–5. (**c**) Potential binding sites of miR-29b-2-5p on the circCacna1c sequence. (**d**) The expression of miR-29b-2-5p in H9c2 cells after knockdown of circCacna1c with/without ISO treatment. One-way ANOVA was used for statistical analysis, *n* = 3. (**e**) Verification of miR-29b-2-5p as sponged targets of circCacna1c by dual-luciferase reporter assay. Left: mutated nucleotides on the binding sites of circCacna1c with miR-29b-2-5p (shown as red letters); Right: the statistical chart of relative luciferase activity. *T*-test was used for statistical analysis, *n* = 3. Data are shown as mean ± SEM. * *p* < 0.05, ** *p* < 0.01. ISO: isoprenaline hydrochloride; WT: wild type; MUT: mutant.

CircCacna1c is mainly located in the cytoplasm, indicating that circCacna1c may impact cardiac hypertrophy by competitively binding miRNA. We then applied TargetScan and miRanda from Arraystar, which predicated five miRNAs that are targeted by circCacna1c, namely miR-135a-2-3p, miR-6914-5p, miR-5126, miR-29b-2-5p, and miR-669a-5p (Figure 4a and Figure S2). The expression of miR-29b-2-5p and miR-669a-5p was examined in hypertrophic mouse heart tissue. The expression of miR-29b-2-5p was downregulated, whereas the expression of miR-669a-5p was not altered (Figure 4b). Therefore, we next further explored the regulatory effect between circCacna1c and miR-29b-2-5p in H9c2 cells and identified two potential binding sites (196bp-219bp and 300bp-327bp) for miR-29b-2-5p on the circCacna1c sequence (Figure 4c). The expression of miR-29b-2-5p was also downregulated in ISO-induced H9c2 cells. Knocked-down circCacna1c increased the expression of miR-29b-2-5p in H9c2 cells (Figure 4d). Finally, we cloned the full circCacna1c sequence and inserted it downstream of a luciferase reporter (circCacna1c-WT) in HEK293T cells. miR-29b-2-5p inhibited luciferase activity compared with NC-mimic. However, the impact was abolished when the miR-29b-2-5p binding site in the circCacna1c was mutated (circCacna1c-MUT, Figure 4e).

### 3.5. Overexpression of miR-29b-2-5p Inhibits Hypertrophic Gene Expression in ISO-Induced H9c2 Cells

**Figure 5:**
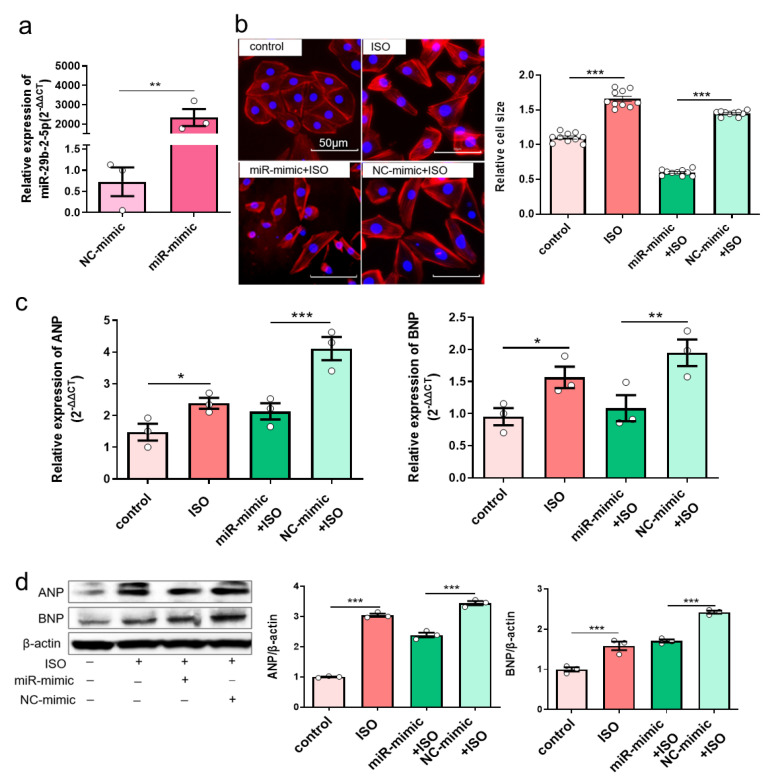
Overexpression of miR-29b-2-5p inhibits hypertrophic gene expression in ISO-induced H9c2 cells. (**a**) MiR-29b-2-5p overexpression efficiency in H9c2 cells transfected with specific miRNA mimic by quantitative real-time polymerase chain reaction assay. *T*-tests were used for statistical analysis, *n* = 3. (**b**) The morphology of ISO-induced H9c2 cells with miR-29b-2-5p overexpression by TRITC-Phalloidin staining assay. The scale bar is 50 μm. One-way ANOVA was used for statistical analysis, *n* = 10. (**c**,**d**) mRNA and protein expression of ANP and BNP in ISO-induced H9c2 cells with overexpression of miR-29b-2-5p, respectively. One-way ANOVA was used for statistical analysis, *n* = 3. Data are shown as mean ± SEM. * *p* < 0.05, ** *p* < 0.01, *** *p* < 0.001. ISO: isoprenaline hydrochloride.

Because of downregulated miR-29b-2-5p in myocardial hypertrophy, we then set to find out whether miR-29b-2-5p affects the expression of hypertrophic genes. MiR-29b-2-5p was overexpressed in H9c2 cells (Figure 5a), which effectively reduced the size of H9c2 cells after being stimulated with 30μM ISO for 36h (Figure 5b). Additionally, the mRNA expression of ANP and BNP was decreased in ISO-induced H9c2 cells with overexpression of miR-29b-2-5p (Figure 5c). In agreement, the protein expression of ANP and BNP was also reduced (Figure 5d), indicating that miR-29b-2-5p overexpression blocks cardiac hypertrophic gene expression.

### 3.6. Silencing circCacna1c Decreases Hypertrophic Gene Expression via miR-29b-2-5p/NFATc1 Axis

**Figure 6:**
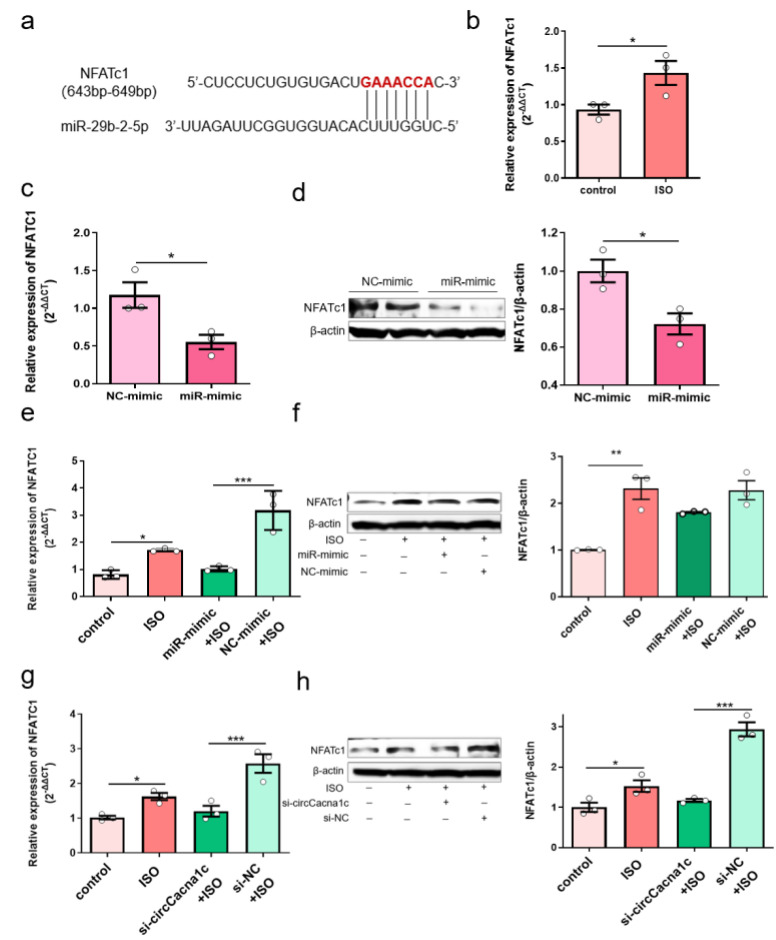
Silencing circCacna1c decreases hypertrophic gene expression via miR-29b-2-5p/NFATc1 axis. (**a**) Potential binding sites of miR-29b-2-5p on the NFATc1 sequence predicted by TargetScan. (**b**) Detection of the expression of NFATc1 mRNA in ISO-induced H9c2 cells by quantitative real-time polymerase chain reaction assay. (**c**,**d**) Detection of mRNA and protein expression of NFATc1 in H9c2 cells with overexpression of miR-29b-2-5p. *T*-test was used for statistical analysis. (**e**,**f**) Detection of the mRNA and protein expression of NFATc1 in ISO-induced H9c2 cells with overexpression of miR-29b-2-5p. (**g**,**h**) Detection of the mRNA and protein expression of NFATc1 in ISO-induced H9c2 cells with knockdown of circCacna1c. One-way ANOVA was used for statistical analysis. Data are shown as mean ± SEM. *n* = 3. * *p* < 0.05, ** *p* < 0.01, *** *p* < 0.001. ISO: isoprenaline hydrochloride.

Further bioinformatic analysis revealed that NFATc1 was a potential target gene for miR-29b-2-5p (Figure S3). We predicted the target genes of miR-29b-2-5p using the miRNA target gene prediction databases and obtained 70 common target genes (Figure S3a). GO enrichment analysis revealed that 16 of these 70 target genes mainly participate in biological processes (*p* < 0.05), including GO: 0007507 (heart development), with 5 enriched genes, namely GYS1, MYOCD, NF1, NFATc1, and TEAD1 (Figure S3b). KEGG pathway enrichment analysis revealed that these 70 target genes were enriched in 3 related signaling pathways, with only the Wnt signaling pathway having statistical significance (*p* < 0.05). The enriched genes included VANGL1, MAPK10, NFATc1, and WNT9A (Figure 3c). The common gene from the GO and KEGG analysis was NFATc1, which we study as the preferred target gene for miR-29b-2-5p action. The 3’UTR of NFATc1 contains one potential target site for miR-29b-2-5p (Figure 6a). Expression of NFATc1 was upregulated in ISO-induced H9c2 cells (Figure 6b). However, the mRNA and protein expressions of NFATc1 were reduced when miR-29b-2-5p was overexpressed (Figure 6c,d). These data suggest that miR-29b-2-5p negatively regulates NFATc1 gene and protein expression in H9c2 cells. Overexpression of miR-29b-2-5p also reduced the mRNA level of NFATc1 in ISO-treated H9c2 cells (Figure 6e), but not the protein expression of NFATc1 (Figure 6f). Silencing circCacna1c increased the expression of miR-29b-2-5p, which decreased mRNA and protein expression of NFATc1 in ISO-induced H9c2 cells (Figure 6g,h). Together, these data suggest that silencing circCacna1c might reduce the expression of NFATc1 through the increase in miR-29b-2-5p expression, thereby reducing the expression of hypertrophic genes.

## 4. Discussion

A growing body of research has revealed that circRNAs are widely found in eukaryotic cells and participate in a variety of biological functions [29,30]. CircRNAs exist in a stable form in the blood, making circRNAs excellent candidate biomarkers in the diagnosis and prognosis of diseases compared to linear RNA [31,32,33], and circRNA could regulate the expression of its host genes [34,35]. However, there are relatively few studies on their functions in the development and progression of pathological cardiac hypertrophy. Here, we have identified novel circRNA associated with cardiac hypertrophy through microarray screening. This novel circRNA, derived from exon two and three of the Cacna1c gene, was named circCacna1c. The expression of circCacna1c was upregulated in ISO-induced hypertrophic mice tissue and H9c2 cells. Knockdown circCacna1c expression abolished ISO-induced hypertrophic growth, along with the mRNA and protein expression of ANP and BNP in H9c2 cells. Mechanistically, we found that circCacna1c targets miR-29b-2-5p/NFATc1 to regulate hypertrophic gene expression in vitro.

It has been shown that circRNAs located in the cytoplasm may act as miRNA sponges, regulating the expression of miRNA-targeted genes [36]. In our study, we found that circCacna1c mainly existed in the cytoplasm. Bioinformatic analysis predicted that circCacna1c binds to five miRNAs, namely miR-135a-2-3p, miR-6914-5p, miR-5126, miR-29b-2-5p, and miR-669a-5p. Mattia Quattrocelli et al. found that overexpression of miR-669a could reduce ANP levels and ameliorate chronic dilated cardiomyopathy in dystrophic mice [37]. Here, we observed no difference in the expression of miR-669a-5p in ISO-induced hypertrophic cardiac tissue. Previously, miR-29c was decreased in transgenic mouse expressing activated calcineurin A in the heart [38], and miR-29a-3p inhibits ET-1-induced cardiomyocyte hypertrophy via inhibiting NFATc4 expression [39]. Here, we show that miR-29b-2-5p was downregulated in mice heart tissue under hypertrophic conditions. Further analysis shows that miR-29b-2-5p was targeted by circCacna1c, indicating that overexpression of miR-29b-2-5p could attenuate ISO-induced pathological hypertrophy. The direct binding of circCacna1c to miR-29b-2-5p was further confirmed by the dual-luciferase reporter assay. MiR-29 has multiple loci (a, b, c), and their mature sequences are mostly the same. As such, we see that miR-29a, miR-29b, and miR-29c can participate in the pathological process of cardiac hypertrophy and can act on the same target (NAFT). However, the presence of several different bases between their sequences may lead to differences in the subtypes of the target genes or modes of action. MiR-29a-3p acts on NAFT4, while our experiment found that miR-29a-2-5p acts on NAFTc1 in cardiac hypertrophy.

The NFATc family is critically involved in cardiac hypertrophy by activating hypertrophic gene expression [40,41,42,43]. While initial research shows that miRNA mainly reduces protein expression by inhibiting translation [44], a later study by David P Bartel et al. shows that miRNA mainly degrades mRNA instead of inhibiting translation to perform its function [45]. Our data showed that overexpression of miR-29b-2-5p significantly inhibited ISO-induced NFATc1 mRNA expression, and its expression level basically returned to the control group level (Figure 6e), while the NFATc1 protein expression level was still much higher than the control group level (Figure 6f), indicating that exogenous miR-29b-2-5p may mainly play a role in degrading target gene mRNA. On the other hand, silencing circCacna1c can restore both ISO-induced NFATc1 mRNA and protein levels to that of the control group (Figure 6h,g), indicating that silencing circRNA indirectly increases endogenous miR-29b-2-5p, which may play a dual role by degrading mRNA and inhibiting protein synthesis. This difference in mechanism suggests the significance of studying circRNA through the ceRNA mechanism, although a certain miRNA directly interacts with target genes. This phenomenon may be a uniqueness of circCacna1c that we have presently discovered, and it is worth further research.

## 5. Conclusions

**Figure 7:**
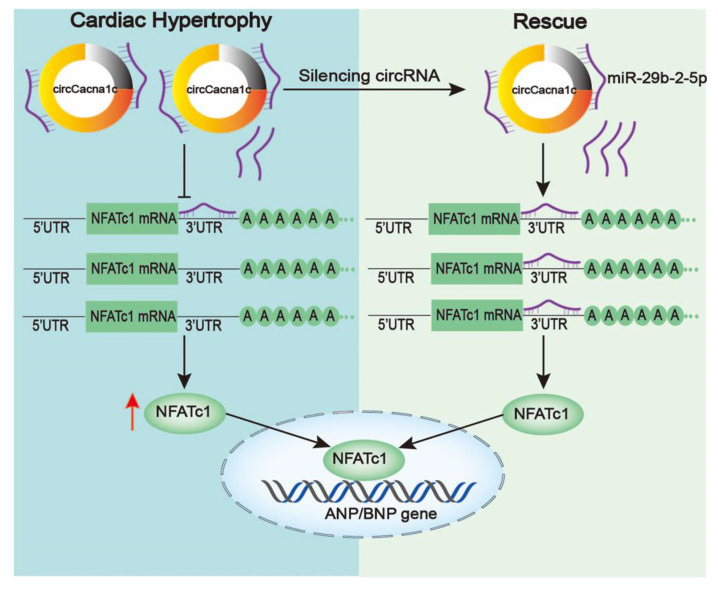
Schematic diagram of the mechanism underlying silencing circCacna1c affects the cardiac hypertrophy by miR-29b-2-5p/NFATc1 axis. Left: circCacna1c expression is upregulated when stimulated by ISO and competitively inhibits the binding of miR-29b-2-5p to NFATc1 mRNA, which increases the expression of NFATc1. Right: free miR-29b-2-5p is induced after silencing circCacna1c, thereby enhancing interaction between miR-29b-2-5p and NFATc1 mRNA, resulting in reduced NFATc1 and hypertrophic gene expression.

In conclusion, our experiments indicate that circCacna1c could be acting as miR-29b-2-5p sponge to regulate NFATc1 in cardiac hypertrophy. The upregulation of circCacna1c promotes the mRNA and expression of NFATc1 by downregulating miR-29b-2-5p in H9c2 cells and mouse hearts treated with ISO. Silencing circCacna1c and the overexpression of miR-29b-2-5p can effectively block cardiac hypertrophy by decreasing the expression of NFATc1. Together, our study reveals a novel paradigm in controlling ISO-induced pathological hypertrophy and dysfunction through a circCacna1c/miR-29b-2-5p/NFATc1 axis (Figure 7). 

Our current experiment was only a preliminary exploration of circCacna1c in vitro. We will conduct further in vivo research to validate the present findings. Moreover, we found hsa_circ_0025016 (homologue of circCacna1c) in circBank. This suggests that we can carry out further corresponding clinical research.

## Data Availability

Data is contained within the article or Supplementary Material.

## Supplementary Materials

The following supporting information can be downloaded at: https://www.mdpi.com/article/10.3390/cells12121667/s1, Table S1: Body weight and heart weight in the model mice; Table S2: The conservation of circCacna1c between mouse and rat species; Table S3: The conservation of circCacna1c between mouse and human species; Figure S1: Haematoxylin and eosin (HE) staining in ISO-induced hypertrophic heart tissues; Figure S2: Potential binding sites of four targeted miRNAs on the circCacna1c sequence; Figure S3: Screening of NFATc1 target gene for miR-29b-2-5p.
