# Supplementary material for: Silencing of circCacna1c Inhibits ISO-Induced Cardiac Hypertrophy through miR-29b-2-5p/NFATc1 Axis
Source: Cells. 2023 Jun 19;12(12):1667. doi: 10.3390/cells12121667 (PMC10297193; doi:10.3390/cells12121667)
Supplement: Supplementary file 1 [file cells-12-01667-s001.zip › cells-2421021-supplementary.pdf]

Supplemental Materials

Table S1. Body weight and heart weight in the model mice.

Groups	BW_i (g)	BWe (g)	HW (mg)	LVW (mg)	HW/BW_e (mg/g)	LVW/BW_e (mg/g)
control (n=3)	18.8 ± 0.8	23.4 ± 0.4	101.2 ± 2.1	74.9 ± 2.3	4.32 ± 0.02	3.20 ± 0.13
ISO (n=3)	18.4 ± 0.4	23.7 ± 0.3	125.0 ± 1.3 ***	95.2 ± 1.0**	5.27 ± 0.12 **	4.02 ± 0.09 **

Note: BW_i: the initial of body weight; BW_e: the end of body weight; HW: heart weight; LVW: left ventricular weight; ISO: isoprenaline hydrochloride. ** $p < 0.01$ v.s. control; *** $p < 0.001$ v.s. control.

Table S2. The conservation of circCacna1c between mouse and rat species.

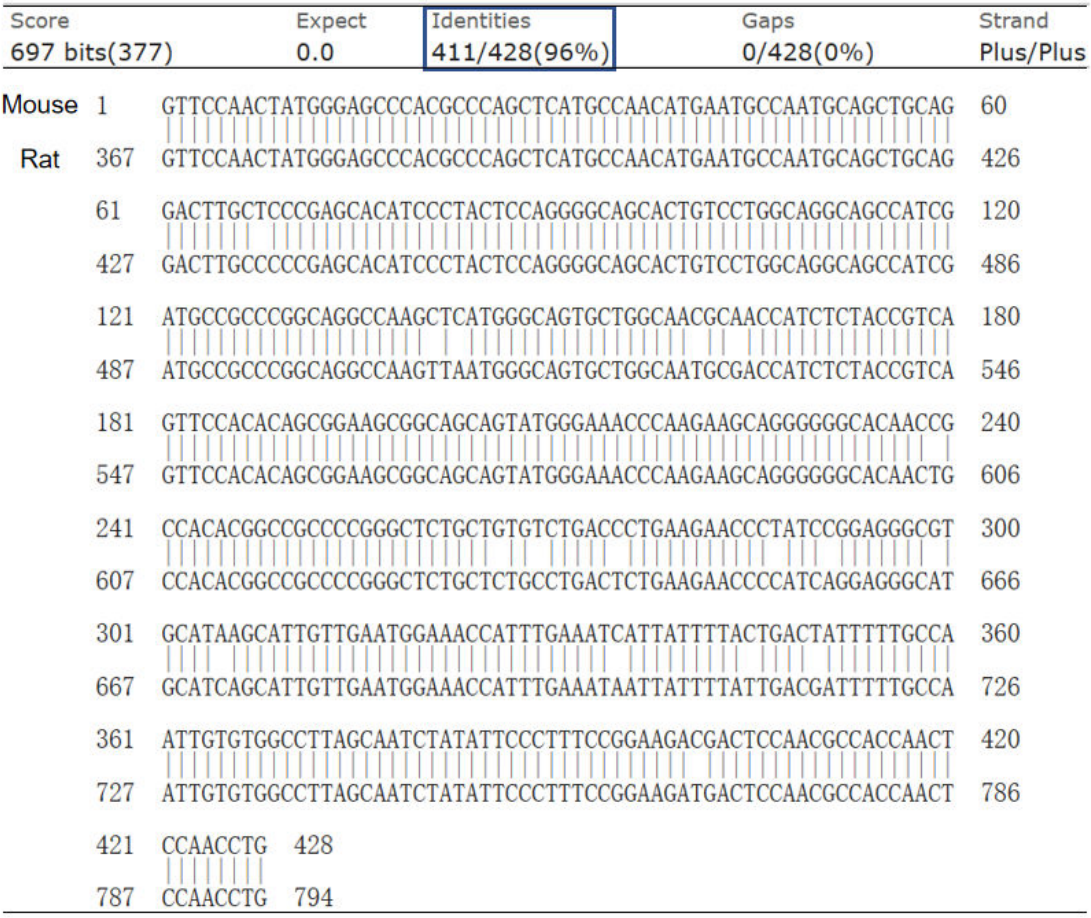

Score	Expect	Identities	Gaps	Strand
697 bits(377)	0.0	411/428(96%)	0/428(0%)	Plus/Plus

```
Mouse 1    GTTCCAACTATGGGAGCCCACGCCCAGCTCATGCCAACATGAATGCCAATGCAGCTGCAG  60
Rat   367  GTTCCAACTATGGGAGCCCACGCCCAGCTCATGCCAACATGAATGCCAATGCAGCTGCAG  426

      61   GACTTGCTCCCGAGCACATCCCTACTCCAGGGGCAGCACTGTCCTGGCAGGCAGCCATCG  120
      427  GACTTGCCCCCGAGCACATCCCTACTCCAGGGGCAGCACTGTCCTGGCAGGCAGCCATCG  486

      121  ATGCCGCCCGGCAGGCCAAGCTCATGGGCAGTGCTGGCAACGCAACCATCTCTACCGTCA  180
      487  ATGCCGCCCGGCAGGCCAAGTTAATGGGCAGTGCTGGCAATGCGACCATCTCTACCGTCA  546

      181  GTTCCACACAGCGGAAGCGGCAGCAGTATGGGAAACCCAAGAAGCAGGGGGGCACAACCG  240
      547  GTTCCACACAGCGGAAGCGGCAGCAGTATGGGAAACCCAAGAAGCAGGGGGGCACAACTG  606

      241  CCACACGGCCGCCCCGGGCTCTGCTGTGTCTGACCCTGAAGAACCCTATCCGGAGGGCGT  300
      607  CCACACGGCCGCCCCGGGCTCTGCTCTGCCTGACTCTGAAGAACCCCATCAGGAGGGCAT  666

      301  GCATAAGCATTGTTGAATGGAAACCATTTGAAATCATTATTTTACTGACTATTTTTGCCA  360
      667  GCATCAGCATTGTTGAATGGAAACCATTTGAAATAATTATTTTATTGACGATTTTTGCCA  726

      361  ATTGTGTGGCCTTAGCAATCTATATTCCCTTTCCGGAAGACGACTCCAACGCCACCAACT  420
      727  ATTGTGTGGCCTTAGCAATCTATATTCCCTTTCCGGAAGATGACTCCAACGCCACCAACT  786

      421  CCAACCTG  428
      787  CCAACCTG  794
```

All experimental protocols were preapproved by the Experimental Animal Ethic Committee of Harbin Medical University (Animal Experimental Ethical Inspection Protocol No. 2009104).

Table S3. The conservation of circCacna1c between mouse and human species.

Score	Expect	Identities	Gaps	Strand
531 bits(287)	6e-148	381/428(89%)	0/428(0%)	Plus/Plus

Mouse	1	GTTCCAACTATGGGAGCCCACGCCCAGCTCATGCCAACATGAATGCCAATGCAGCTGCAG	60
Human	626	GTTCCAACTATGGGAGCCCACGCCCCGCCCATGCCAACATGAATGCCAATGCGGCAGCGG	685
	61	GACTTGCTCCCGAGCACATCCCTACTCCAGGGGCAGCACTGTCCTGGCAGGCAGCCATCG	120
	686	GGCTGGCCCCTGAGCACATCCCCACCCCGGGGGCTGCCCTGTCGTGGCAGGCGGCCATCG	745
	121	ATGCCGCCCGGCAGGCCAAGCTCATGGGCAGTGCTGGCAACGCAACCATCTCTACCGTCA	180
	746	ACGCAGCCCGGCAGGCTAAGCTGATGGGCAGCGCTGGCAATGCGACCATCTCCACAGTCA	805
	181	GTTCCACACAGCGGAAGCGGCAGCAGTATGGGAAACCCAAGAAGCAGGGGGGCACAACCG	240
	806	GCTCCACGCAGCGGAAGCGGCAGCAATATGGGAAACCCAAGAAGCAGGGCAGCACCACGG	865
	241	CCACACGGCCGCCCCGGGCTCTGCTGTGTCTGACCCTGAAGAACCCTATCCGGAGGGCGT	300
	866	CCACACGCCCGCCCCGAGCCCTGCTCTGCCTGACCCTGAAGAACCCCATCCGGAGGGCCT	925
	301	GCATAAGCATTGTTGAATGGAAACCATTTGAAATCATTATTTACTGACTATTTTTGCCA	360
	926	GCATCAGCATTGTCGAATGGAAACCATTTGAAATAATTATTTACTGACTATTTTTGCCA	985
	361	ATTGTGTGGCCTTAGCAATCTATATTCCCTTTCCGGAAGACGACTCCAACGCCACCAACT	420
	986	ATTGTGTGGCCTTAGCGATCTATATTCCCTTTCCAGAAGATGATTCCAACGCCACCAATT	1045
	421	CCAACCTG	428
	1046	CCAACCTG	1053

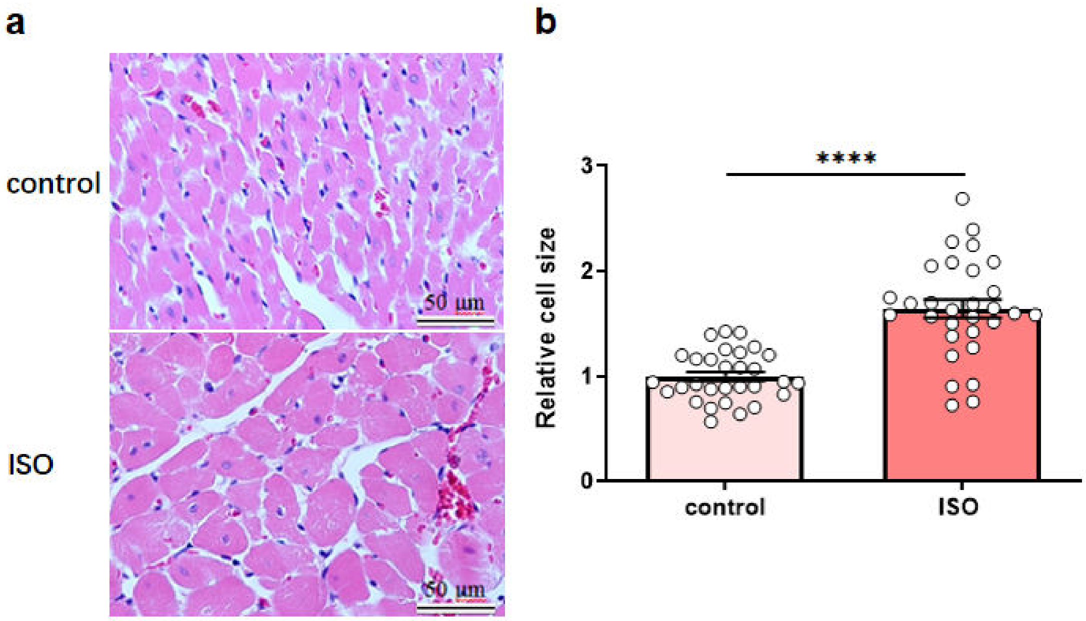

Figure S1. Haematoxylin and eosin (HE) staining in ISO-induced hypertrophic heart tissues. (**a**) Typical original image of HE staining in mouse heart tissue after ISO treatment. The scale bar is 50 μm. (**b**) Statistical graph of relative myocardial cell size from control and ISO groups. 30 cells from 3 mice for each group. ISO: isoprenaline hydrochloride.

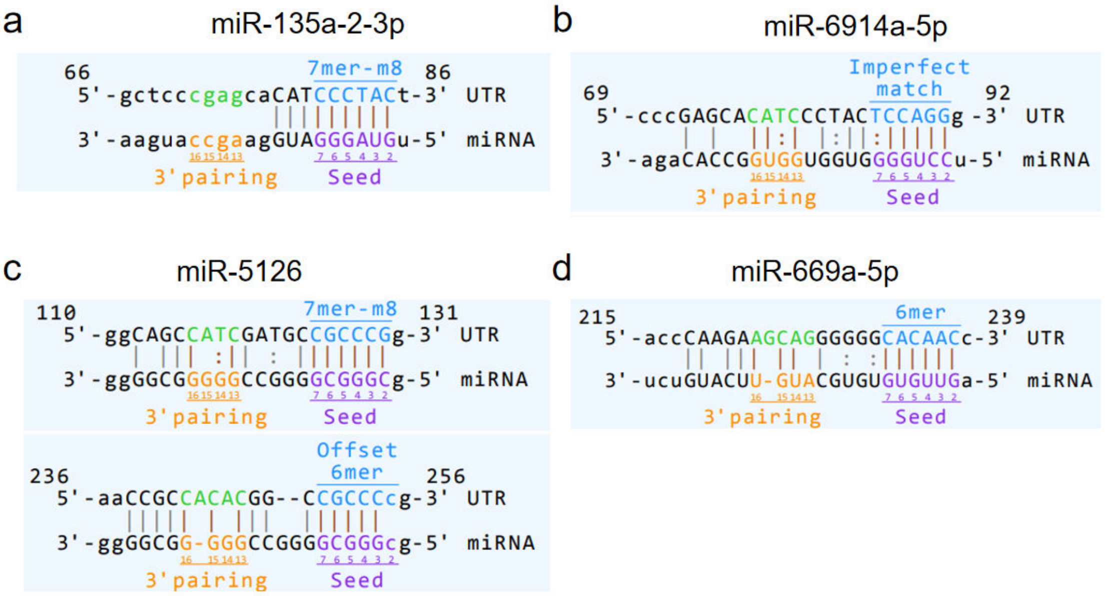

Figure S2. Potential binding sites of four targeted miRNAs on the circCacna1c sequence. (**a**) miR-135-2-3p. (**b**) miR-6914a-5p. (**c**) miR-5126. (**d**) miR-669a-5p.

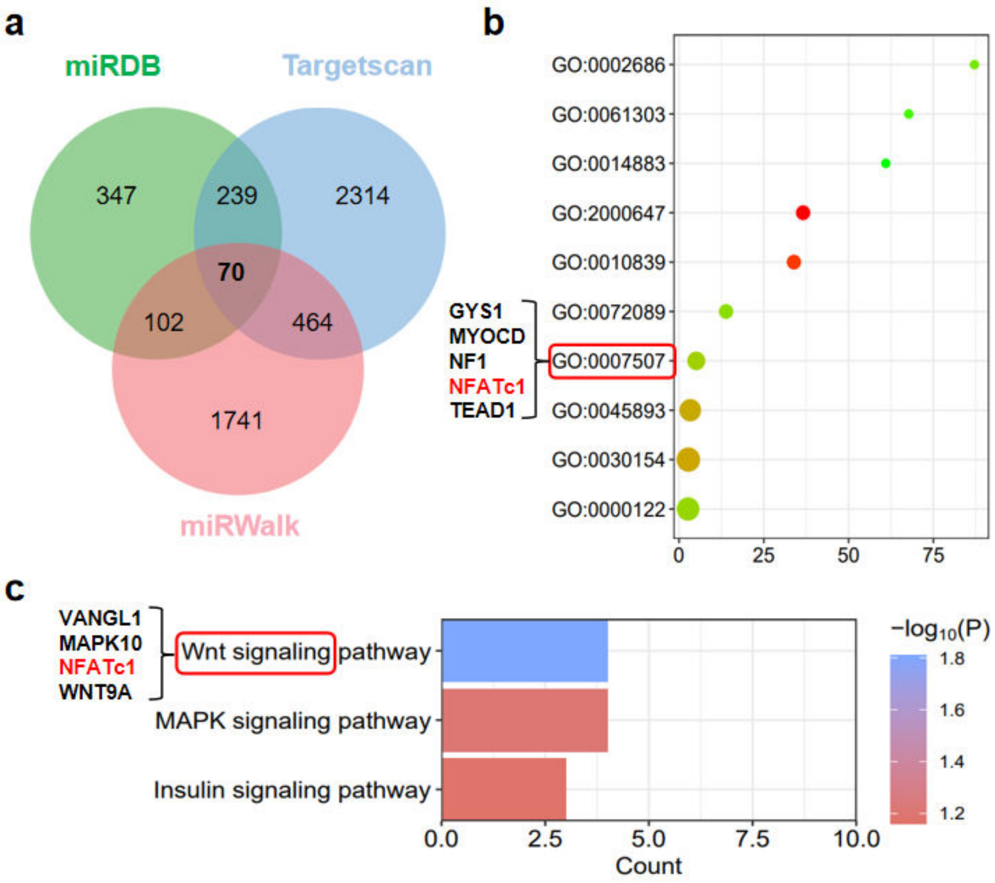

Figure S3. Screening of NFATc1 target gene for miR-29b-2-5p. (**a**) Predicting the target genes of miR-29b-2-5p using TargetScan, miRDB, and miRWalk databases. (**b**) GO enrichment analysis for the 70 common target genes of miR-29b-2-5p using using DAVID (https://david.ncifcrf.gov/) and Metascape websites (http://metascape.org/). (**c**) KEGG pathway enrichment analysis for the 70 common target genes of miR-29b-2-5p using using DAVID and Metascape websites.
